# Supplementary material for: Early Palaeozoic ocean anoxia and global warming driven by the evolution of shallow burrowing
Source: Nat Commun. 2018 Jul 2;9:2554. doi: 10.1038/s41467-018-04973-4 (PMC6028391; doi:10.1038/s41467-018-04973-4)
Supplement: Supplementary file 1 — Supplementary Information [file 41467_2018_4973_MOESM1_ESM.pdf]

Supplementary information for:

Evolution of shallow burrowing triggered long-term ocean anoxia and global warming in the early Palaeozoic

van de Velde et al.

Supplementary Note 1: Proxies for ocean redox state

Supplementary Figure 1 documents three different redox proxies: Fe speciation, Cerium anomaly (Ce_{anom}) and Molybdenum abundances[1–3]. The principles of Fe redox proxies are well-documented (see refs[4,5] and references therein). Supplementary Figure 1a shows a schematic picture of the dominant ocean redox state throughout the early Palaeozoic, based on a recent compilation[3]. Following generally increased oxygenation in the Ediacaran, the Cambrian explosion is coincident with an increase in anoxia, which persists until the Ordovician-Silurian.

The abundance of Cerium relative to other rare earth elements (in this case $Ce_n / (\mathrm{Pr}_n^2 / Nd_n)$, where n indicates normalisation to post-Archean Australian shale (PAAS)) can be used as a measure for oxidative Ce removal. See ref. [1] for more information. Depletion of Ce (indicating greater Ce removal, and thus more oxygenated seawater) then leads to Ce_{anom} values < 1. The Ce_{anom} curve indicates an increase in seawater oxygenation in the lead up to the Cambrian explosion, followed by a relative decrease throughout the early Palaeozoic (Supplementary Figure 1b).

The final redox proxy shown in Supplementary Figure 1c is Mo concentration. Although less clearly linked to oxygenation than Cerium and Fe speciation, the abundance of Mo (and uranium) indicates that the ocean may have become deoxygenated between the Ediacaran and Ordovician.

All of the redox proxies indicate temporal and spatial variability, and also suffer from a sparse record. Although oxygen concentrations appear to be highly dynamic, there exists a general consensus on the overall oxygenation trend for the end of the Proterozoic and early Palaeozoic, which is supported by a number of works: the end of the Ediacaran was marked by an increase in oxygen[6–10], while the early Cambrian exhibit a general increase in ocean anoxia[7,11–13].

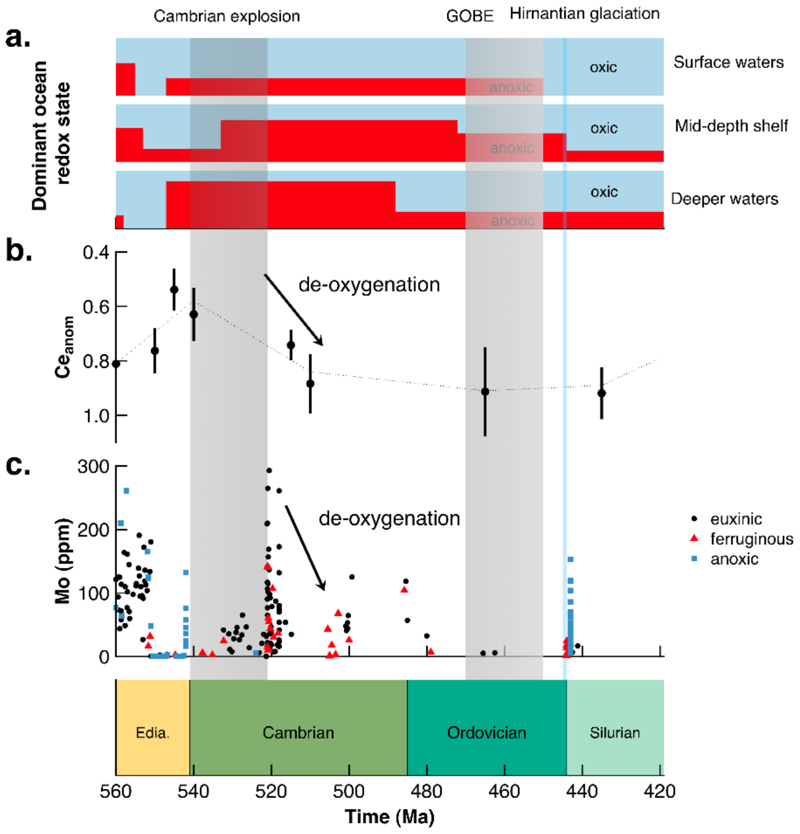

Supplementary Figure 1: compilation of different ocean redox proxies. (a) evolution of the dominant ocean redox state, based on the Fe redox proxy compilation of ref [3]. (b) summary of Cerium anomaly (Ce_{anom}) over time, as compiled by ref [1]. Error bars represent 1 standard deviation (+/- 1 S.D.). (c) summary of Mo concentrations, as compiled by ref [2]. Shaded areas refer to the Cambrian explosion (540 Ma – 521 Ma) and the Great Ordovician Biodiversification Event (GOBE; 470 Ma – 440 Ma).

Supplementary Note 2: Atmospheric oxygen and marine sulphate concentrations.

Supplementary Figure 2 shows the model output of atmospheric O_2 and marine $[SO_4]$ for the baseline model[14] and the two scenarios considered in the main manuscript. The COPSE model is originally intended to evaluate the global biogeochemical trends of the Phanerozoic[15], and this is especially apparent when one tries to model atmospheric oxygen and marine sulphate concentrations before the advent of bioturbation. In the baseline model, bioturbation is implicitly assumed to be at its maximum value throughout the whole modelled time-period, and during the Cambrian, atmospheric O_2 is ~0.2 PAL, while $[SO_4]$ ~10 mM. No model run shows a large impact on the fraction of sulphur buried as (f_{pyr}). Introducing bioturbation in all scenarios results in very high ambient levels of atmospheric O_2 before the evolution of bioturbation, as well as near present day marine sulphate concentrations ($[SO_4]$ ~20 mM). This appears unlikely when considering the available evidence. In order to fully employ COPSE in a pre-bioturbation world, it is necessary to re-evaluate some of the model processes. Whilst beyond the scope of the current manuscript, there are a number of considerations here: for example, in the Neoproterozoic ocean, which was rich in reduced species such as Fe^{2+}, and to a lesser extent HS^-[7,16,17], anoxygenic photosynthesis was probably a much more important photosynthetic pathway than in the present day ocean, and while this would contribute to organic carbon burial, it would not directly produce O_2[18], potentially leading to the overestimation observed in our model. Also, a consideration of methane cycling would likely reduce the predictions for atmospheric O_2.

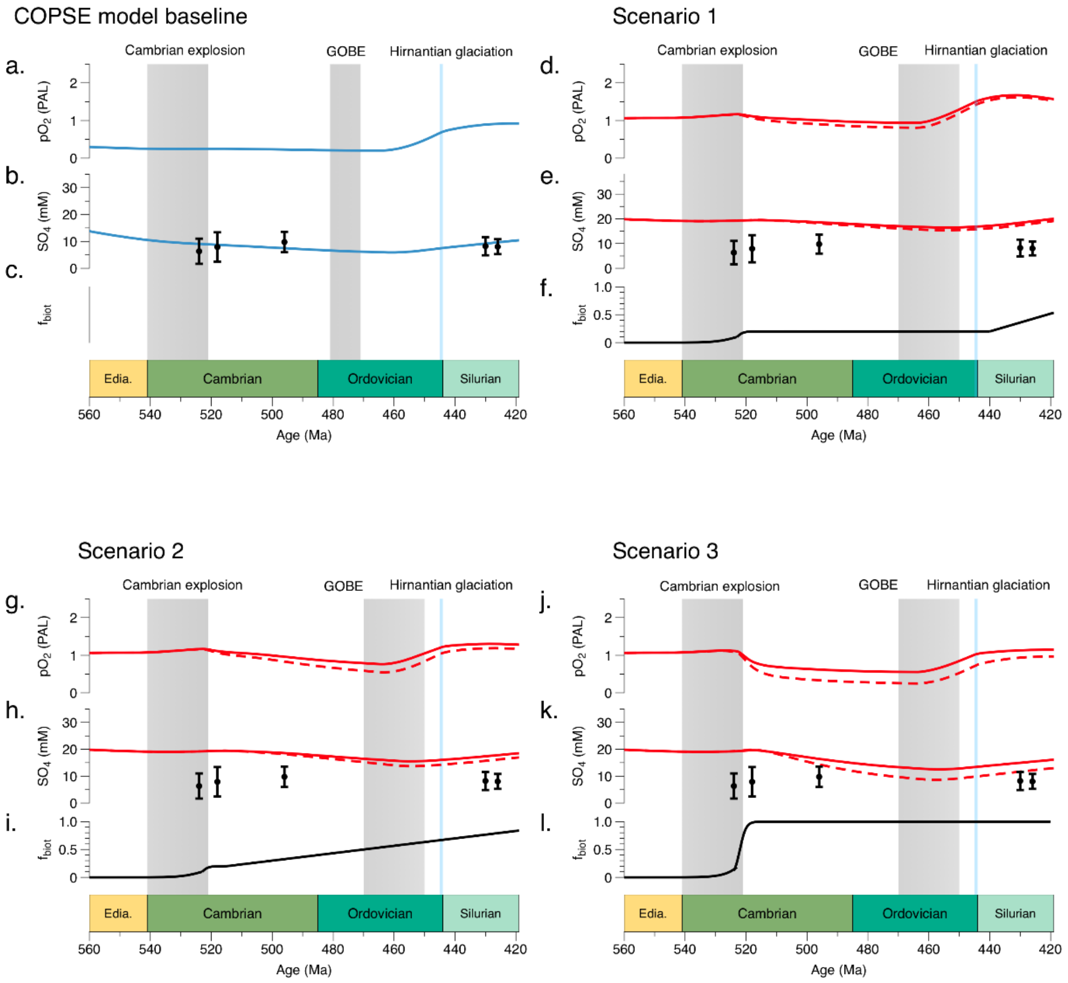

Supplementary Figure 2: COPSE model without and with the addition of the evolution of bioturbation. Scenario 1 shows the effect of bioturbation delayed until the Silurian (see panels c, f, i), Scenario 2 gradually increases the importance of bioturbation across the Palaeozoic and Scenario 3 assumes that effective bioturbation was established in the Cambrian (see panels f, i, l). (a),(d),(g),(j) Atmospheric O_2. (b),(e),(h),(k) Sulphate concentration of seawater. Error bars represent 1 standard deviation (+/- 1 S.D.) Dashed lines show the model output with anoxia feedback on bioturbation turned off, full lines show model output with anoxia feedback on bioturbation.

Supplementary Note 3: Model sensitivity to individual effects of bioturbation.

Supplementary Figures 3, 4 and 5 show bioturbation evolution Scenarios 1, 2 and 3 respectively. These figures are split to show the individual effects of bioturbation on marine organic carbon burial (mocb), marine organic phosphorus burial (mopb) and marine pyrite sulphur burial (mpsb). Both the coupling between the C_{org}:P_{org} ratios of buried organic matter and bioturbation, and the direct effect of carbon mineralization have strong effects on the model predictions. The magnitude of the changes is sensitive to values chosen for $CB_{prebiot}$, CP_{biot} and CP_{lam}, but the qualitative trajectory is consistent between runs. Bioturbation-enhanced sulphur cycling has little effect on the model outcomes. Increasing $SC_{prebiot}$ decreases $\delta^{34}S_{SO4}$ by limiting the burial rate of pyrite. However, the negative feedbacks in the model limit the effects on ocean sulphate concentration: reducing pyrite burial ultimately increases anoxia, which promotes a compensatory increase in pyrite burial.

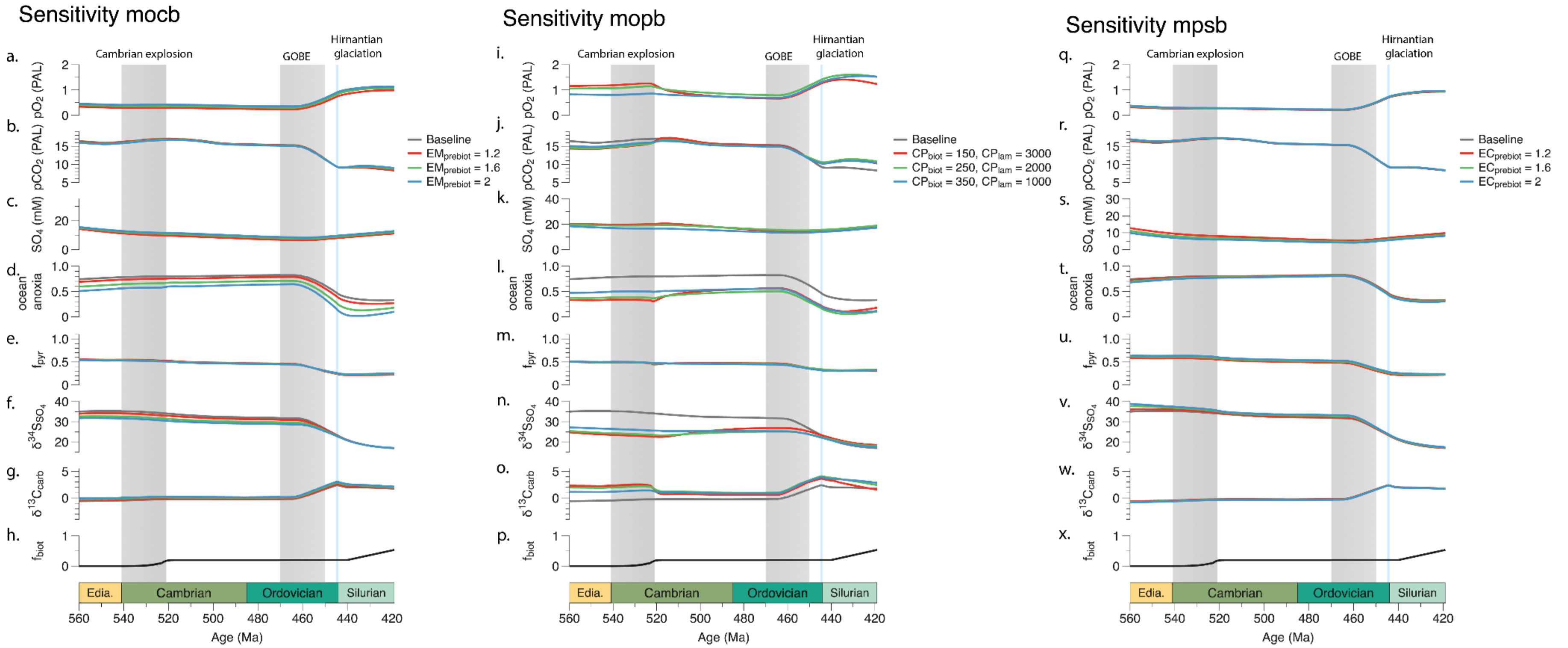

Supplementary Figure 3: sensitivity test of model adaptations of the impact of bioturbation on the burial of organic carbon (mocb, left), the C:P ratio of the buried organic carbon (mopb, middle) and the burial of marine pyrite sulphur (mpsb, right) (see text for details). Biogeochemical response to bioturbation scales with burrowing depth (Scenario 1 as described in the main text). No anoxia feedback was included in the model run in order to distinguish the isolated effect of the investigated parameters.

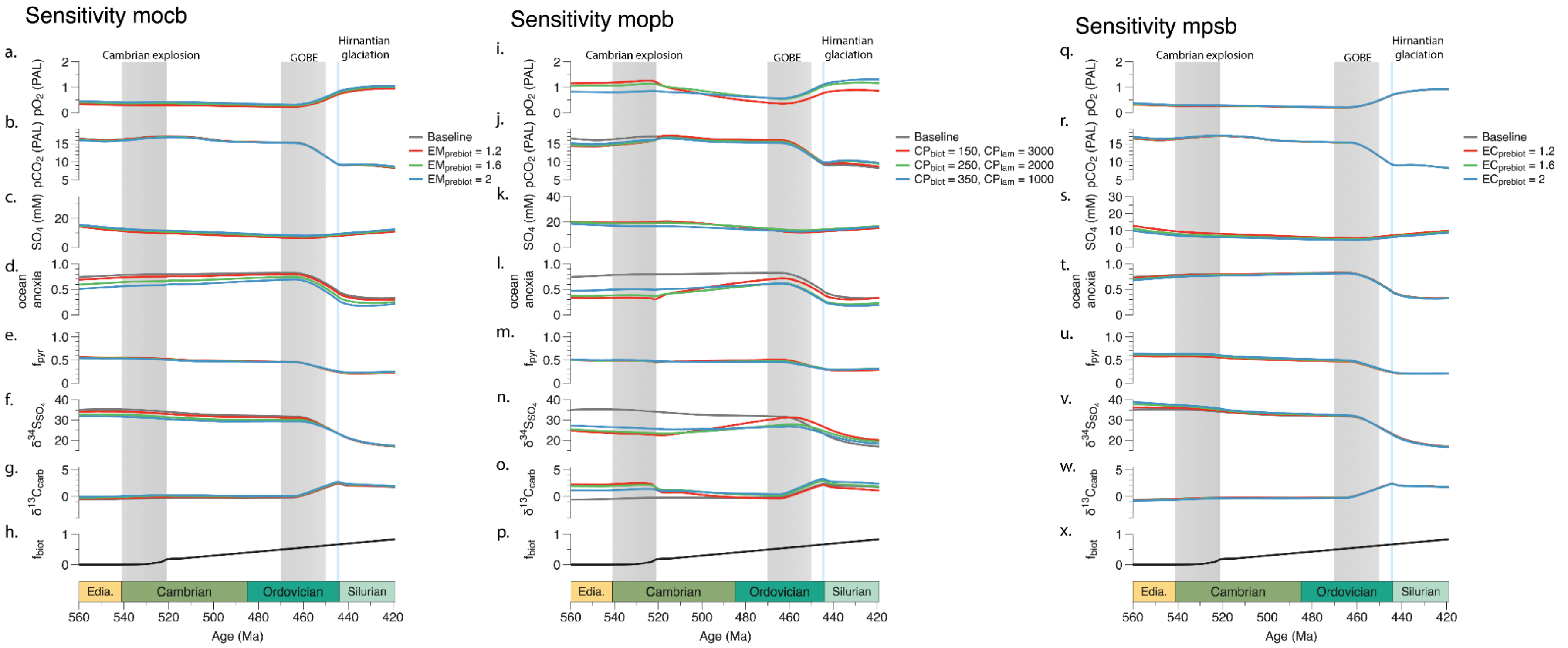

Supplementary Figure 4: sensitivity test of model adaptations of the impact of bioturbation on the burial of organic carbon (mocb, left), the C:P ratio of the buried organic carbon (mopb, middle) and the burial of marine pyrite sulphur (mpsb, right) (see text for details). Biogeochemical response to bioturbation is non-linear and spatial extent gradually increases across the Palaeozoic (Scenario 2 as described in the main text). No anoxia feedback was included in the model run in order to distinguish the isolated effect of the investigated parameters.

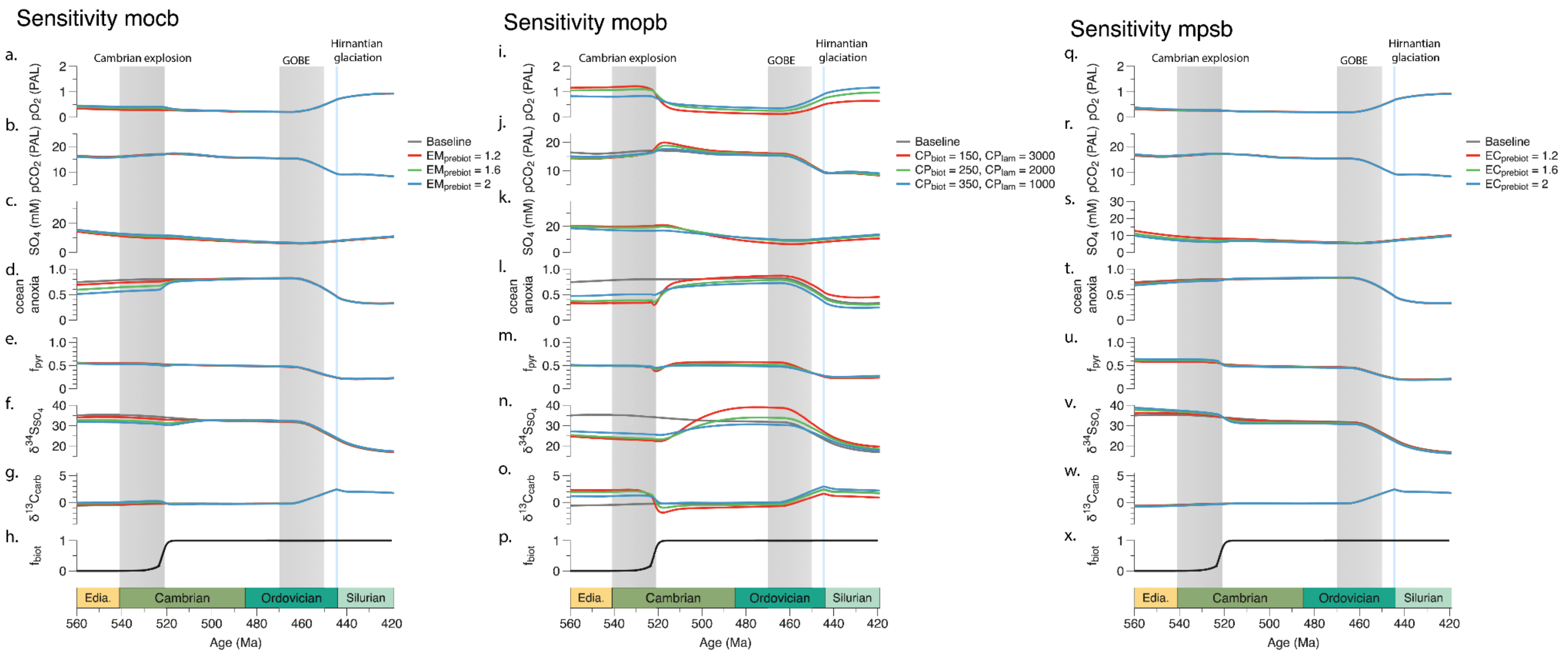

Supplementary Figure 5: sensitivity test of model adaptations of the impact of bioturbation on the burial of organic carbon (mocb, left), the C:P ratio of the buried organic carbon (mopb, middle) and the burial of marine pyrite sulphur (mpsb, right) (see text for details). Biogeochemical response to bioturbation is non-linear (Scenario 3 as described in the main text). No anoxia feedback was included in the model run in order to distinguish the isolated effect of the investigated parameters.

References

1. Wallace, M. W. *et al.* Oxygenation history of the Neoproterozoic to early Phanerozoic and the rise of land plants. *Earth Planet. Sci. Lett.* **466,** 12–19 (2017).
2. Chen, X. *et al.* Rise to modern levels of ocean oxygenation coincided with the Cambrian radiation of animals. *Nat. Comm.* 6 (2015). doi: 10.1038/ncomms8142
3. Sperling, E. a. *et al.* Statistical analysis of iron geochemical data suggests limited late Proterozoic oxygenation. *Nature* **523,** 451–454 (2015).
4. Poulton, S. W. & Canfield, D. E. Ferruginous Conditions: A Dominant Feature of the Ocean through Earth's History. *Elements* **7,** 107–112 (2011).
5. Lyons, T. W. & Severmann, S. A critical look at iron paleoredox proxies: New insights from modern euxinic marine basins. *Geochim. Cosmochim. Acta* **70,** 5698–5722 (2006).
6. Canfield, D. E., Poulton, S. W. & Narbonne, G. M. Late-Neoproterozoic deep-ocean oxygenation and the rise of animal life. *Science* **315,** 92–95 (2007).
7. Canfield, D. E. *et al.* Ferruginous conditions dominated Neoproterozoic Deep-Water Chemistry. *Science (80-.).* **321,** 949–952 (2008).
8. Chen, X. *et al.* Rise to modern levels of ocean oxygenation coincided with the Cambrian radiation of animals. *Nat. Commun.* **6,** 7142 (2015).
9. Fike, D. a, Grotzinger, J. P., Pratt, L. M. & Summons, R. E. Oxidation of the Ediacaran ocean. *Nature* **444,** 744–7 (2006).
10. Pogge von Strandmann, P. A. E. *et al.* Selenium isotope evidence for progressive oxidation of the Neoproterozoic biosphere. *Nat. Commun.* **6,** 10157 (2015).
11. Dahl, T. W. *et al.* Devonian rise in atmospheric oxygen correlated to the radiations of terrestrial plants and large predatory fish. *Proc. Natl. Acad. Sci.* **107,** 17911–17915 (2010).
12. Zhuravlev, A. Y. & Wood, R. A. Anoxia as the cause of the mid Cambrian extinction event. *Geology* **24,** 311–314 (1996).
13. Saltzman, M. R., Edwards, C. T., Adrain, J. M. & Westrop, S. R. Persistent oceanic anoxia and elevated extinction rates separate the Cambrian and Ordovician radiations. *Geology* **43,** 807–811 (2015).
14. Lenton, T. M. *et al.* Earliest land plants created modern levels of atmospheric oxygen. *Proc. Natl. Acad. Sci. U. S. A.* **113,** 9704–9709 (2016).
15. Bergman, N. M., Lenton, T. M. & Watson, A. J. COPSE: A new model of

biogeochemical cycling over phanerozoic time. *Am. J. Sci.* **304,** 397–437 (2004).

16. Johnston, D. T. *et al.* An emerging picture of Neoproterozoic ocean chemistry: Insights from the Chuar Group, Grand Canyon, USA. *Earth Planet. Sci. Lett.* **290,** 64–73 (2010).
17. Guilbaud, R., Poulton, S. W., Butterfield, N. J., Zhu, M. & Shields-zhou, G. a. A global transition to ferruginous conditions in the early Neoproterozoic oceans. *Nat. Geosci.* 6–11 (2015). doi:10.1038/NGEO2434
18. Johnston, D. T., Wolfe-Simon, F., Pearson, A. & Knoll, A. H. Anoxygenic photosynthesis modulated Proterozoic oxygen and sustained Earth's middle age. *Proc. Natl. Acad. Sci.* **106,** 16925–16929 (2009).
